# Brain-Targeted 5-ALA-CAT Liposomes (BACL) Alleviate Hypoxia and Enhance Photodynamic Therapy in a Murine Glioblastoma Flank Xenograft Model via Angiopep-2-Mediated Targeting

**DOI:** 10.3390/pharmaceutics18070777

**Published:** 2026-06-25

**Authors:** Qian Zhang, Yuhang Li, Jiahui Zhang, Xuewen Zhao, Danlu Li, Wenting Zhao, Xin Hai, Xin Chen, Xinlei Yang, Jingxin Gou, Chunpeng Zhang, Xing Tang, Yilei Zhao

**Affiliations:** 1 Department of Pharmacy, The First Affiliated Hospital of Harbin Medical University, Harbin 150001, China; 19829136728@163.com (Q.Z.); li1324307746@163.com (Y.L.); 15164485820@163.com (J.Z.); zhaoxw5571@163.com (X.Z.); yxlidanlu@126.com (D.L.); zwting1986@163.com (W.Z.); hai_xin@163.com (X.H.); zhangcp1796@163.com (C.Z.); 2 Department of Neurosurgical Laboratory, The First Affiliated Hospital of Harbin Medical University, Harbin 150001, China; chenxin_tracy@yeah.net; 3 Heilongjiang Province Key Laboratory of Precision Pharmaceutical Research, The First Affiliated Hospital of Harbin Medical University, Harbin 150001, China; 4 Animal Laboratory Center, The First Affiliated Hospital of Harbin Medical University, Harbin 150001, China; xinleiyang@hotmail.com; 5 Department of Pharmaceutics, School of Pharmacy, Shenyang Pharmaceutical University, Shenyang 110016, China; jxgou_syphu@163.com

**Keywords:** glioblastoma, 5-Aminolevulinic acid, catalase, liposomes, photodynamic therapy

## Abstract

**Background/Objectives:** Glioblastoma multiforme (GBM) treatment is limited by tumor hypoxia and poor specificity of therapeutic agents. To address these challenges, we developed brain-targeted liposomes co-encapsulating 5-aminolevulinic acid (5-ALA) and catalase (CAT), termed brain-targeted 5-ALA-CAT liposomes (BACL), which were surface-modified with the Angiopep-2 ligand to enhance blood–brain barrier penetration and achieve multimodal therapy combining targeted delivery and oxygen generation. **Methods:** BACL was prepared and characterized. Tumor targeting was verified by flow cytometry and in vivo imaging. In vitro antitumor activity was evaluated by wound-healing assay, colony formation assay, live/dead staining, MTT assay, and Western blotting. In vivo efficacy, apoptosis, and safety were assessed in a subcutaneous xenograft model. Transcriptome sequencing and qRT-PCR were employed to identify molecular mechanisms and novel targets. **Results:** BACL exhibited favorable physicochemical properties (size: 122.4 nm, PDI: 0.189, zeta potential: −12.3 mV) and spherical morphology as observed by TEM, with encapsulation efficiencies of 51.2% for 5-ALA and 43.8% for CAT. Compared with unmodified 5-ALA, BACL increased the cellular uptake efficiency by 1.6-fold in glioma cells while maintaining catalytic stability for sustained oxygen generation. In vitro experiments demonstrated that BACL significantly inhibited glioma cell migration, colony formation, and cell viability, and induced apoptosis. In a subcutaneous xenograft tumor model, BACL-mediated photodynamic therapy (PDT) achieved a tumor growth inhibition rate of 52%, with apoptosis induction via regulation of Bcl-2, Bax, and p53 expression, and no obvious toxicity to major organs was observed. Transcriptomic analysis combined with qRT-PCR validation revealed that BACL activates multiple antitumor signaling pathways, including targeted inhibition of IL-10 and CXCL13 to disrupt cytokine–receptor interactions, as well as coordinated regulation of S100A3 and IGSF-9 expression to suppress glioma progression. **Conclusions:** These multimodal actions enhanced PDT efficacy while remodeling the tumor microenvironment. Our findings position BACL as a promising therapeutic platform integrating targeted delivery, hypoxia alleviation, and immunomodulation for GBM therapy.

## 1. Introduction

Glioblastoma multiforme (GBM) is the most prevalent and aggressive primary brain tumor, with an annual incidence of approximately 6 cases per 100,000 individuals [1]. Despite advances in surgery, radiotherapy, and temozolomide-based chemotherapy, GBM remains highly invasive and intrinsically resistant to cytotoxic therapies. Consequently, median overall survival is limited to 12–15 months after diagnosis [2]. These challenges underscore the urgent need for innovative multimodal strategies to modulate the tumor microenvironment and improve GBM therapy.

Photodynamic therapy (PDT) has emerged as a promising non-invasive treatment for GBM [3]. The prodrug 5-aminolevulinic acid (5-ALA) is converted in tumor mitochondria to the photosensitizer protoporphyrin IX (PpIX). Upon irradiation at 630–635 nm, PpIX generates singlet oxygen (^1^O_2_) and other reactive oxygen species (ROS) [4]. Unlike DNA-damaging therapies, PDT offers selective tumor accumulation, low off-target toxicity, and a reduced risk of resistance—advantages that are particularly valuable for preserving neural function in brain tumors [5,6].

However, clinical application of 5-ALA PDT faces two interconnected challenges. First, the oxygen-dependent mechanism creates a therapeutic dilemma: ROS generation consumes O_2_, worsening pre-existing hypoxia and establishing a vicious cycle that limits PDT efficacy [7,8,9]. Second, the blood–brain barrier (BBB) severely restricts 5-ALA delivery, leading to insufficient PpIX accumulation in GBM and limiting its surgical utility for low-grade tumors [10,11].

Current strategies to alleviate tumor hypoxia include vascular remodeling, hyperbaric oxygen therapy, and enzymatic decomposition of H_2_O_2_ [12,13]. However, vascular remodeling is hampered by pre-existing vessel damage, and hyperbaric oxygen suffers from poor oxygen diffusion into hypoxic zones. Nanocatalytic approaches that exploit endogenous H_2_O_2_ have therefore attracted considerable interest [14,15,16,17]. Catalase (CAT), an enzyme that efficiently converts H_2_O_2_ into O_2_, is particularly promising for modulating hypoxic conditions [3,18].

Concurrently, ligand-conjugated nanocarriers including polymeric micelles and liposomes have improved the delivery of 5-ALA [19]. Among these, Angiopep-2 functionalized liposomes enable BBB penetration via low-density lipoprotein receptor-mediated transcytosis, achieving enhanced GBM targeting [20,21,22].

**Figure 1:**
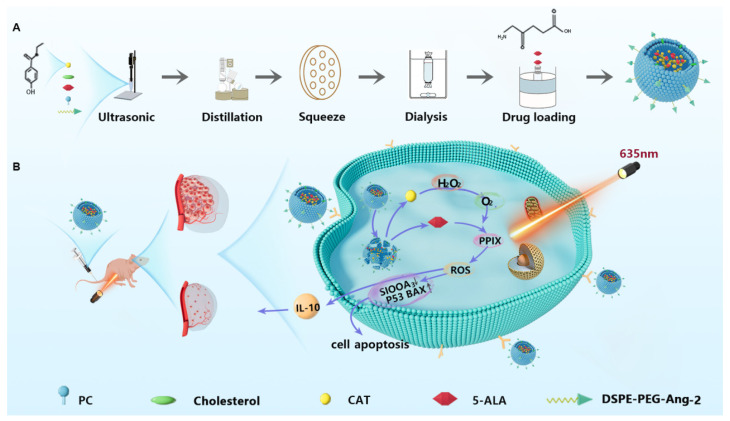
Schematic illustration of the preparation and therapeutic mechanism of BACL. (**A**) The preparation process and structural composition of a novel BACL. (**B**) BACL significantly enhanced the targeted uptake of 5-ALA by GBM cells, improved the hypoxic microenvironment of these cells, and increased the efficacy of 5-ALA photodynamic treatment for GBM.

Building on these advancements, we developed a dual-functional nanoplatform termed BACL. BACL co-encapsulates 5-ALA and CAT in Angiopep-2-modified liposomes, thereby addressing both drug delivery barriers and tumor hypoxia (Figure 1). This design yields three key improvements: (1) alleviation of tumor hypoxia via CAT-mediated O_2_ generation; (2) enhanced 5-ALA bioavailability through liposomal protection; and (3) improved BBB penetration and tumor accumulation via Angiopep-2-mediated targeting. Our results demonstrate that BACL enhances PDT efficacy by coordinating photosensitizer delivery and oxygen supply, offering a promising strategy for precision GBM therapy.

## 2. Materials and Methods

### 2.1. Materials

5-Aminolevulinic acid hydrochloride (5-ALA, purity ≥ 99%) was purchased from Shanghai Yuanye Biotechnology Co., Ltd. (Shanghai, China). Catalase (specific activity ≥ 2000 U/mg protein) was obtained from Beijing Solarbio Technology Co., Ltd. (Beijing, China). DSPE-PEG_2000_-Angiopep-2 (DSPE-PEG_2000_-Ang-2) and cholesterol were procured from Shanghai Advanced Vehicle Technology Co., Ltd. (Shanghai, China). Soybean phosphatidylcholine (Lipoid PC50, PC content ~ 50% and Lipoid PC70, PC content ~ 70%), purified soybean phosphatidylcholine (Lipoid S100, PC content ≥ 94%) and egg yolk phospholipids (Lipoid E80, PC content ~ 80%) were purchased from Shanghai Dongsheng Pharmaceutical Technology Co., Ltd. (Shanghai, China). 3-(4,5-Dimethylthiazol-2-yl)-2,5-diphenyltetrazolium bromide (MTT) was purchased from Sigma Aldrich (St. Louis, MO, USA). Dulbecco’s modified Eagle’s medium (DMEM) was acquired from Gibco (Grand Island, NY, USA), and fetal bovine serum (FBS) was from Dalian Meilun Biotechnology Co., Ltd. (Dalian, China). The Annexin V-FITC Apoptosis Detection Kit was obtained from Amy Jet Technology Co., Ltd. (Wuhan, China), the Reactive Oxygen Species Assay Kit (DCFH-DA) from Beyotime Biotechnology Co., Ltd. (Shanghai, China), and the Image-iT Green Hypoxia Reagent from Thermo Fisher Scientific (Waltham, MA, USA).

### 2.2. Animals

Female BALB/c nude mice (4–6 weeks old, weighing 18–22 g, SPF, immunodeficient) and male Sprague–Dawley (SD) rats (6–8 weeks old, weighing 230–270 g) were provided by Beijing Vitacol River Laboratory Animal Technology Co., Ltd. (Beijing, China). The experimental animals were raised in a standard specific pathogen-free (SPF) environment, with the environmental temperature controlled at 22 ± 2 °C, relative humidity at 50 ± 5%, and a light cycle of 12 h/12 h. After grouping, the animals were placed in a sterile ventilated cage and were allowed to freely access standard feed and sterilized drinking water. Rats were anesthetized via intraperitoneal injection of 2.5% tribromoethanol. All operations have been approved by the Experimental Animal Ethics Committee of the First Affiliated Hospital of Harbin Medical University (Ethical Approval Number: 2024021, approval date: 6 September 2024).

### 2.3. Preparation and Lyophilization Stability Study of BACL Liposomes

The preparation of BACL involves two steps. Firstly, Ang2-modified liposomes (BCL) are prepared through reverse evaporation combined with extrusion method. Then, BACL is obtained by incubating with 5-ALA solution in a water bath. In short, PC70, cholesterol, DSPE-PEG_2000_, and DSPE-PEG_2000_-Ang2 (molar ratio 64:14:1:2) were dissolved in chloroform/ether (2:3). Under ice-bath conditions, PBS (pH 7.4) containing 0.15% (*w*/*v*) CAT was added dropwise, followed by sonication at 40 W for 5 min. The organic solvent was removed by rotary evaporation at 37 °C. The resulting suspension was extruded sequentially through 200 nm and 100 nm polycarbonate membranes (three times each). Free CAT was removed by dialysis (MWCO 100kDa, Millipore, Burlington, MA, USA, 24 h) to obtain brain-targeted CAT liposomes (BCL).

To achieve efficient encapsulation of 5-ALA into liposomes, a pH-gradient remote loading method was employed, a strategy originally established by Barenholz and co-workers for loading amphipathic weak acids into preformed liposomes [23]. BCL was mixed with 5-ALA (final concentration 0.15%, *w*/*v*), and the external pH was adjusted to 6.0, 7.0, and 8.0, respectively. The mixture was incubated at 40 °C for 25 min, followed by dialysis (MWCO 100 kDa) to remove unloaded 5-ALA, yielding BACL. BACL was stored at 4 °C and used within 10 days. The lyoprotective effect of trehalose was evaluated at five concentrations (0.5%, 1%, 3%, 5%, and 10%) using the particle size, PDI, and appearance of the formulation before and after lyophilization as indicators. Lyophilization consisted of pre-freezing (ramping from room temperature to −40 °C over 2 h and holding for 4 h), evacuation to 350 mTorr for 30 min, and stepwise drying ramps to −20 °C over 2 h (held for 3 h), to −10 °C over 1 h (held for 3 h), to 0 °C over 2 h (held for 3 h), and to 20 °C over 2 h (held for 2 h). The optimal formulation was selected based on these criteria. Furthermore, the stability of the optimal formulation was assessed by monitoring changes in particle size and PDI over a period of one month.

### 2.4. Liposome Characterization

Dynamic light scattering (Malvern Zetasizer Nano ZS, Malvern Panalytical, Malvern, UK) was used to measure the hydrodynamic diameter. Samples were diluted 20-fold with deionized water and equilibrated at 25 °C for 5 min; measurements were performed at 37 °C in triplicate. Morphology of BACL was observed by transmission electron microscopy (TEM, JEOL, Tokyo, Japan) after negative staining with 2% phosphotungstic acid. Briefly, 5 μL of BACL (0.1 mg/mL) was placed on a carbon-coated copper grid, stained for 30 s, air-dried, and excess stain was removed with filter paper. The entrapment efficiency (*EE*%), loading capacity (*LC*%) and in vitro release profile of 5-ALA and CAT were determined by ultrafiltration centrifugation (Millipore, Burlington, MA, USA; 100 kDa MWCO, 3000 r/min, 15 min) combined with UV-vis spectroscopy (Unico Instrument Co., Ltd., Shanghai, China; 340 nm for 5-ALA; 595 nm for CAT after Bradford assay using bovine serum albumin as standard) (Figure S1). The ultrafiltration retentate was washed once with 0.5 mL PBS and centrifuged again, and the combined filtrate was used to quantify free drugs. The serum stability of BACL was systematically evaluated by incubating it in 50% fetal bovine serum (FBS) at 37 °C for 24 h. Storage stability was assessed at 4 °C over 14 days by monitoring particle size, PDI, and *EE*% daily. *EE*% and *LC*% were calculated as:

$$EE\left(\%\right)=\frac{Totaldrug\;-\;Freedrug}{Totaldrug}\times 100\%\;\;\;$$

$$LC\left(\%\right)=\frac{Amount\;of\;drug\;within\;the\;formulation}{Total\;Mass\;of\;the\;formulation}\times 100\%$$

### 2.5. Evaluation of Catalase Activity

The catalytic activities of BACL and free CAT were measured using the standard Góth’s method [24]. Briefly, H_2_O_2_ (50 mM) was co-incubated with BACL or free CAT at 37 °C. The reaction was then terminated by adding ammonium molybdate and cooling the mixture on an ice bath to 25 °C. Unreacted H_2_O_2_ was evaluated by UV-vis spectroscopy at 400 nm, based on its primary yellow complexes with ammonium molybdate. As a control, H_2_O_2_ without CAT or BACL was maintained at 37 °C and subsequently reacted with ammonium molybdate at 25 °C. The *relative catalytic activity* was calculated using the following formula:

$$Relative\;catalytic\;activity(\%)=\frac{Absorbance\;of\;H_{2}O_{2}\;with\;PBS\;-\;Absorbance\;of\;H_{2}O_{2}\;with\;BACL}{Absorbance\;of\;H_{2}O_{2}\;with\;PBS\;-\;Aabsorbance\;of\;H_{2}O_{2}\;with\;free\;CAT}\times 100\%$$

To evaluate the resistance of embedded CAT and free CAT in BACL and ACL to proteinase K, both were co-incubated with active proteinase K (0.5 mg/mL, experimental group) or without proteinase K (negative control group) at 37 °C, respectively. Samples were collected at 0, 0.25, 0.5, 1, 2, 4 and 8 h, and the CAT catalytic activity was evaluated using Góth’s method. Taking the 0 h without proteinase K activity as 100%, calculate the relative activity at each time point to evaluate the stability of CAT.

### 2.6. Cell Culture and Uptake

To evaluate the cellular uptake of 5-ALA and its liposomal formulations under hy-poxic conditions, the following experiment was performed. Human GBM cells (U251), BV2 and human umbilical vein endothelial cells (HUVEC) were purchased from Procell Life Science & Technology (Wuhan, China) and cultured in DMEM supplemented with 10% fetal bovine serum and 1% penicillin/streptomycin at 37 °C in 5% CO_2_. Cells were seeded in 6-well plates at 1 × 10^5^ cells per well. After complete adhesion, plates were trans-ferred to a three-gas incubator (Thermo FORMA 3131, Thermo Fisher Scientific, Waltham, MA, USA) and preconditioned under hypoxia (1% O_2_, 5% CO_2_, 37 °C) for 24 h. Subsequently, the cells were further incubated for 4 h under the same hypoxic conditions with the following treatments: control (medium only), free 5-ALA, AL, ACL, and BACL, each at an equivalent 5-ALA concentration of 6 μg/mL. After treatment, cells were detached with trypsin, collected by centrifugation, washed three times with PBS, and resuspended in 0.5 mL PBS. The intracellular protoporphyrin IX (PpIX) fluorescence was measured by flow cytometry using a 405 nm excitation laser and a 660/20 nm emission filter.

### 2.7. ROS Generation in U251 Cells

To evaluate intracellular ROS production in U251 cells following various treatments, cells were seeded in 6-well plates at a density of 1 × 10^5^ cells per well. Cells were then incubated with control (medium only), free 5-ALA, AL, ACL, and BACL, all at an equivalent 5-ALA concentration of 6 μg/mL, in the presence of 50 μM H_2_O_2_ for 4 h. After treatment, each well was irradiated with a 635 nm laser at 0.3 W/cm^2^ for 3 min. ROS levels were immediately measured by flow cytometry using the fluorescent probe DCFH-DA (Beyotime, Shanghai, China) according to the manufacturer’s protocol. Data were analyzed and presented as the mean fluorescence intensity (MFI).

### 2.8. Hypoxia Monitoring

U251 cells were seeded in 6-well plates (1 × 10^5^ cells/well) and cultured under hypoxia (37 °C, 1% O_2_, 5% CO_2_) for 24 h. Upon reaching 70% confluence, the medium was replaced with fresh medium containing 2 μmol/L Image-iT™ Green Hypoxia Reagent and incubated for 30 min. After removing the reagent, cells were washed once with PBS (1 mL/well) to remove extracellular probe. Cells were then divided into four groups: normoxic control (NC, 21% O_2_, 5% CO_2_, 37 °C), hypoxic control (Ctrl, 1% O_2_), ACL, and BACL (interventions defined elsewhere). All groups received 6 μg/mL, 5-ALA in fresh medium and were incubated for 4 h under their respective oxygen conditions (NC in normoxia; Control, ACL, and BACL in hypoxia).

### 2.9. Western Blot Analysis

After treatment (5-ALA 6 μg/mL, H_2_O_2_ 50 μM, 4 h), hypoxic U251 cells were irradiated (635 nm, 0.3 W/cm^2^, 5 min) and incubated overnight under hypoxia without medium change. Cells were lysed in RIPA buffer with protease/phosphatase inhibitors. Protein concentration was determined by BCA assay. Equal amounts (30 μg) were separated by SDS-PAGE, transferred to PVDF membranes, and blocked. Membranes were incubated overnight at 4 °C with primary antibodies against BAX, Bcl-2, p53, HIF-1α (all 1:1000), and β-actin (1:2000), followed by secondary antibody (1:2000, 1 h, RT). Bands were visualized using ECL.

### 2.10. Cytotoxicity Assay

U251 cells were seeded in 96-well plates (2 × 10^3^ cells/well) and cultured under hypoxia (37 °C, 1% O_2_, 5% CO_2_) for 24 h. The medium was then replaced with fresh hypoxic medium containing serial dilutions of each test compound (5-ALA, AL, ACL, BACL; 5-ALA at 0–6 μg/mL, and AL, ACL, BACL at molar equivalent concentrations to 5-ALA; blank liposome at matched lipid concentration), plus 50 μM H_2_O_2_ for all groups except the untreated control. Cells were incubated under hypoxia for 4 h. After removing the drug-containing medium, fresh medium was added, and each well was irradiated with a 635 nm laser (0.3 W/cm^2^, 5 min). Cells were then incubated for another 24 h under hypoxia. Subsequently, 20 μL of MTT solution (5 mg/mL in PBS) was added to each well and incubated for 4 h. The supernatant was removed, and the formazan crystals were dissolved in 100 μL DMSO. Absorbance was measured at 490 nm using a microplate reader, with background subtraction at 630 nm.

### 2.11. Clonogenic Assay

U251 cells were seeded at 1000 cells/well in 6-well plates and cultured under hypoxia for 24 h. The medium was replaced with DMEM containing the indicated drugs (5-ALA, AL, ACL and BACL; 5-ALA at 6 μg/mL, others at equimolar concentrations) plus 50 μM H_2_O_2_, followed by hypoxic incubation for 3 d. On day 4, each well was irradiated with a 635 nm laser (0.3 W/cm^2^) for 3 min. After refreshing with drug-free DMEM, cells were cultured under hypoxia for another 7 d. Then, cells were washed with PBS, fixed with 4% paraformaldehyde for 10 min, air-dried, stained with 0.1% crystal violet for 10 min, and photographed.

### 2.12. Apoptosis Analysis

U251 cell apoptosis was assessed by Annexin V-FITC/PI staining and flow cytometry. Logarithmic phase cells were seeded in 6-well plates (1 × 10^5^ cells/well) and pre cultured under hypoxia (37 °C, 1% O_2_, 5% CO_2_) for 24 h. Groups included: blank control, H_2_O_2_ alone (50 μM), and drugs plus 50 μMH_2_O_2_ (5-ALA, AL, ACL, and BACL; each at 6 μg/mL 5-ALA equivalent). After replacing the medium with drug-containing DMEM, cells were incubated under continued hypoxia for 4 h. The medium was then replaced with fresh DMEM, and each well was irradiated with a 635 nm laser (0.3 W/cm^2^) for 3 min. Following irradiation, cells were cultured in drug-free DMEM under hypoxia for another 20 h to allow apoptosis. Cells were collected, washed, and resuspended in Annexin V binding buffer. Annexin V-FITC and PI were added, incubated for 20 min at room temperature in the dark, and analyzed by flow cytometry.

### 2.13. Live and Dead Cell Staining

U251 cells (5 × 10^3^/well, 96-well) were hypoxic-preconditioned (37 °C, 1% O_2_, 5% CO_2_, 24 h), treated with control, 5-ALA, AL, ACL and BACL (6 μg/mL 5-ALA) under hypoxia for 4 h, irradiated (635 nm, 0.3 W/cm^2^, 3 min), and post-incubated under hypoxia for 4 h. The cells were stained for 0.5 h using the Calcein/PI Cell Viability and Cytotoxicity Assay Kit (Beyotime) (Calcein-AM 2 μM, PI 4.5 μM) at 37 °C in the dark, washed, and imaged (three fields/well). Live/dead cells were counted using Image J software (v1.54f) to calculate dead cell percentage. Three independent experiments were performed with triplicate wells.

### 2.14. Scratch Healing Experiment

U251 cells (5 × 10^5^/well, 6-well) were grown to 80% confluence, serum-starved (12 h), scratched (200 µL tip), and washed. Hypoxic treatment with control, 5-ALA, AL, ACL and BACL (6 μg/mL 5-ALA) for 4 h, then replaced with serum free medium and cultured under hypoxia for 24 h. Images at 0, 12, 24 h. The *relative migration rate* was calculated using the following formula:

$$Relative\;migration\;rate\left(\%\right)=\frac{Initial\;area\;-\;Area\;at\;t}{Initial\;area}\times 100\%\;$$

### 2.15. Assessing the Blood–Brain Barrier Penetration and PDT Apoptosis

An in vitro blood–brain barrier (BBB) model was established using the Transwellmetho [25,26]. In this model, type I collagen was coated on the lower chamber, and hCMEC/D3 cells were cultured in the upper chamber until tight junctions were formed. The model was used for experimental studies when the trans endothelial electrical resistance (TEER) value exceeded 90–130 Ω·cm^2^. U251 cells were pre-seeded in the lower compartment and cultured in a hypoxic incubator (37 °C, 1% O_2_, 5% CO_2_) for 24 h. Subsequently, 5-ALA, AL, ACL, and BACL (all at a 5-ALA concentration of 6 μg/mL) were added to the upper chamber, followed by continued culture for 12 h. Analysis was then performed using flow cytometry.

Following establishment and treatment of the blood–brain barrier model as described above, the drug-containing medium was replaced with fresh serum-free medium, and the system was exposed to light irradiation (635 nm, 0.3 W/cm^2^). Cells were then detached, collected, washed with PBS, and pelleted by centrifugation. The cell pellets were resuspended in Annexin V-FITC binding buffer and stained with propidium iodide (PI) for 20 min. Apoptosis was subsequently quantified via flow cytometry.

### 2.16. Hemolytic Test

Red blood cells (RBCs) collected from SD rats were washed three times with PBS and diluted to 2% (*v*/*v*). Serial dilutions of Lip, ACL, and BACL (10, 20, 40, 80, 160 μg/mL) were mixed with equal volumes of 2% RBC suspension. PBS and distilled water served as negative (0% hemolysis) and positive (100% hemolysis) controls, respectively. After incubation at 37 °C for 2 h, the mixtures were centrifuged at 3000× *g* for 10 min. The absorbance of the supernatant was measured at 540 nm. A rate < 5% was considered non-hemolytic. Experiments were performed in triplicate. The *hemolysis rate* was calculated using the following formula:

$$Hemolysis\;rate\left(\%\right)=\frac{A_{sample}-A_{PBS}}{A_{distilled\;water}-A_{PBS}}\times 100\%$$

### 2.17. In Vivo Distribution Experiment (In Vivo Imaging)

The animals received intravenous tail vein injections of DiO-labeled ACL or BACL (equivalent to 5-ALA at 40 mg/kg, based on equimolar 5-ALA content), or free DiO dye as a control (*n* = 3 per group). At 6 h post-injection, mice were anesthetized with 1.5–2% isoflurane in oxygen (0.5 L/min) and imaged using a small animal live imaging system (Bruker, Billerica, MA, USA). Excitation and emission wavelengths were set at 484 nm and 501 nm. Fluorescence intensity in regions of interest (ROIs) was quantified as average radiance (photons/s/cm^2^/sr) using the instrument software, after subtracting background autofluorescence.

### 2.18. In Vivo Therapy

U251 cells (3 × 10^6^ cells/mL in 100 μL PBS) were injected subcutaneously into the right flank of each mouse. This T-cell-deficient strain supports human tumor xenografts, where tumor volume and weight serve as primary endpoints for quantifying drug efficacy.

Mice that met the inclusion criteria (tumor formation and tumor volume reaching 100 mm^3^) were included in the analysis. When tumor volumes reached approximately 100 mm^3^, the mice were randomly assigned to five groups (*n* = 6 per group): saline, 5-ALA, AL, ACL, and BACL. All treatments were administered via tail vein injection every other day at an equivalent 5-ALA dose of 40 mg/kg. Six hours post-injection, the tumor site was irradiated with a 635 nm laser (0.3 W/cm^2^, 5 min). Body weight was recorded every two days. After five treatment cycles, mice were sacrificed, and tumor tissues along with major organs (heart, liver, spleen, lungs, kidneys) were collected.

For biosafety assessment, organ sections were stained with H&E. Immunohistochemical staining (anti-p53, anti-Bcl-2, anti-Bax) was performed on tumor sections to evaluate apoptosis. *Tumor volume* was calculated as:

$$Tumor\;volume=\frac{{Width}^{2}\;\times \;Length}{2}$$

The *tumor inhibition rate (TIR)* was calculated based on the isolated tumor weight to evaluate the antitumor efficacy. The formula is as follows:

$$TIR\left(\%\right)=\frac{W_{control}\;\times {\;W}_{treatment}}{W_{control}}\times 100\%$$

where *W_control_* is the mean tumor weight of the control group, and *W_treatment_* is the mean tumor weight of each treatment group.

### 2.19. RNA Sequencing and Data Analysis

GBM tissues were collected from U251 xenografts in nude mice treated with AL and ACL (*n* = 3 per group). RNA isolation, quality control, library construction, and sequencing were carried out by Shanghai OE Biotech Co., Ltd. (Shanghai, China) (https://www.oebiotech.com/, accessed on 1 June 2024). Genes were classified as differentially expressed genes (DEGs) based on the criteria of *p*-value < 0.05 and |log_2_FC| ≥ 1. Volcano plots were generated from all expressed genes. GO (BP/CC/MF), Kyoto Encyclopedia of Genes and Genomes (KEGG), and WikiPathways enrichment were performed using cluster Profiler. protein–protein interaction (PPI) networks were visualized with Cytoscape (v3.9.1). Gene Set Enrichment Analysis (GSEA) was conducted using the cluster Profiler package.

### 2.20. Quantitative Real-Time PCR

Total RNA was extracted from tumor tissues using TRIZOL (Beyotime, Shanghai, China). RNA purity (A260/280: 1.8–2.0; A260/230 ≥ 2.0) and integrity (1% agarose gel) were assessed. 1.0 μg RNA was reverse transcribed using a cDNA synthesis kit (Roche, Basel, Switzerland) with a mix of oligo dT and random hexamers (42 °C, 60 min). The qRT-PCR was performed on an ABI 7500 Fast system (Applied Biosystems, Foster City, CA, USA) using SYBR Green Master Mix. Each 20 μL reaction contained 0.4 μM primers and 2 μL cDNA; cycling: 95 °C 2 min; 40 cycles of 95 °C 15 s, 60 °C 1 min. GAPDH was the internal control, and relative expression was calculated by the formula 2^−ΔΔCt^. All reactions were run in triplicate, with no-template and no-reverse-transcription controls. Primer sequences are listed in Supplementary Table S1.

### 2.21. Statistical Analysis

All experiments were performed in at least three independent replicates, and data are presented as mean ± SD. Statistical analysis was conducted using GraphPad Prism 9.3.1. Curve fitting for the release study was performed using Origin 2021 v9.8.0.200. Differences among multiple groups were analyzed using one-way ANOVA followed by Tukey’s post hoc test. A *p*-value < 0.05 was considered statistically significant (* *p* < 0.05, ** *p* < 0.01, *** *p* < 0.001, ns, not significant, *p* > 0.05).).

## 3. Results

### 3.1. Optimal Prescription and Characterization of BACL

**Figure 2:**
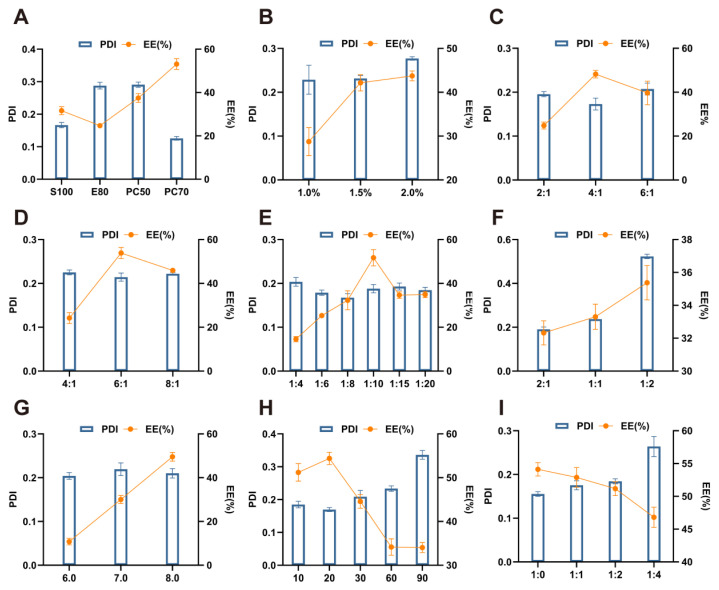
Preparation process screening of liposome. (**A**) Selection of phospholipid types such as S100, E80, PC50 and PC70. (**B**) Investigation of PC70 content (**C**) Investigation of the ratio of cholesterol to PC70. (**D**) Investigation of the Ratio of DSPE-PEG_2000_. (**E**) Investigation of the ratio of drug to lipid. (**F**) Investigation of drug–enzyme ratio. (**G**) Investigation of pH value of the external water item. (**H**) Incubation time investigation. (**I**) targeting ligand molar ratio (DSPE-PEG_2000_: DSPE-PEG_2000_-Ang-2). (Mean ± SD, *n* = 3).

Various formulation parameters, including phospholipid type (S100, E80, PC50, and PC70), phospholipid-to-cholesterol ratio, drug-to-lipid ratio, drug-to-enzyme ratio, incubation pH, and targeting ligand molar ratio, were systematically optimized (Figure 2A–I). The optimal formulation was selected based on appropriate particle size, low polydispersity index (PDI < 0.2), and high encapsulation efficiency.

**Figure 3:**
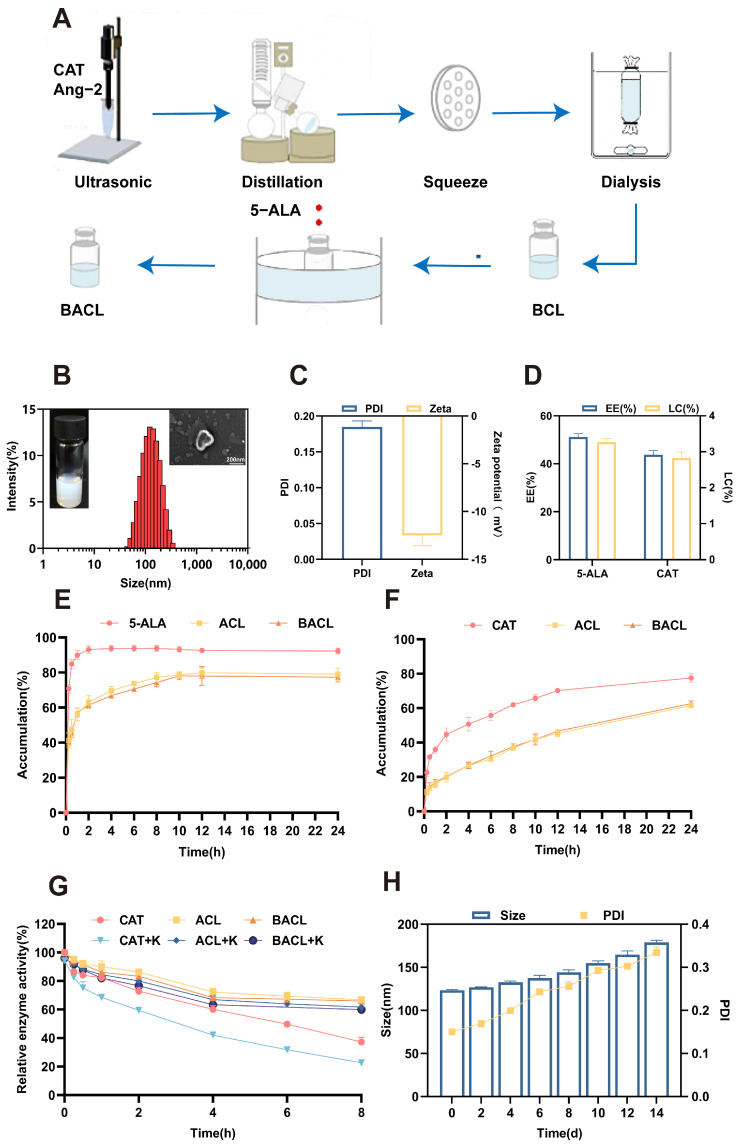
Characterization and stability of BACL. (**A**) Schematic diagram of the liposome preparation process using the reverse evaporation–extrusion method. (**B**) Particle size of BACL detected by dynamic light scattering and TEM imaging, Scale bar, 200 nm. (**C**) Zeta potential and PDI of the liposome. (**D**) Encapsulation efficiency (*EE*) and drug loading (*DL*) of BACL (MWCO 100 kDa). (**E**) Release curve of 5-ALA in liposomes. (**F**) Release curve of CAT in liposomes. Data points represent the mean percentage of drug released. (**G**) The relative enzymatic activity changes of free catalase in the presence or absence of proteinase K. (**H**) Stability of BACL upon storage at 4 °C for 14 days, monitored by changes in particle size and polydispersity index (PDI). (Mean ± SD, *n* = 3).

Using the optimized conditions, BACL was prepared via the reverse-phase evaporation method followed by extrusion (Figure 3A). The resulting BACL appeared as a translucent, milky white suspension exhibiting the typical blue opalescence characteristic of nanoscale liposomes. Transmission electron microscopy revealed spherical vesicles, and dynamic light scattering measurements showed a mean diameter of 122.4 ± 0.24 nm, a zeta potential of −12.3 ± 1.12 mV, and a PDI of 0.189 ± 0.014 (Figure 3B,C). The absolute value of zeta potential reflects the surface charge density of the nanoparticles; the observed negative charge confers sufficient electrostatic repulsion to stabilize the liposomes, thereby mitigating aggregation and potentially prolonging systemic circulation time while enhancing tumor-targeted accumulation [27]. The encapsulation efficiencies and drug loading capacities of 5-ALA and CAT in BACL were determined to be 51.2%/3.3% and 43.8%/2.8%, respectively (Figure 3D).

The lyoprotective effect of trehalose was investigated across concentrations ranging from 0.5% to 10%. The results showed that as the trehalose concentration increased, the appearance of the lyophilized product gradually improved: slight crystal precipitation was observed at 0.5%, partial caking/aggregation at 1%, bottle shrinkage/collapse at 3% and 5%, and a porous and loose structure was formed at 10%. Both the particle size and PDI of the reconstituted formulation showed a decreasing trend with increasing trehalose concentration, indicating that a higher amount of trehalose enhances its lyoprotective effect and effectively maintains the stability of the lipid bilayer structure (Table S2). After optimization of the lyophilization process, the particle size of the formulation changed by less than 10% and the PDI remained below 0.26 during one month of storage at room temperature (25 °C) (Table S3). These results confirm that the addition of trehalose effectively inhibits particle aggregation during lyophilization and significantly improves the storage stability of BACL.

Our results demonstrate that the particle size and polydispersity index (PDI) remain largely stable within the first 6 h of incubation, maintained at approximately 122 nm and 0.181, respectively. Beyond this period, a modest increase in both parameters was observed. At the 12-h time point, the particle size increased to 152 nm and the PDI rose to 0.306. Notably, both parameters plateaued between 12 and 24 h, indicating that no progressive aggregation occurred during the later stage of incubation (Supplementary Figure S1).

The in vitro release profiles of 5-ALA and CAT from BACL were characterized using a dynamic dialysis method (Figure 3E,F and Figure S2). Background subtraction using blank liposomes eliminates interference from the release medium and excipients. Free 5-ALA showed a rapid burst release, reaching approximately 90% at 2 h and 92.3% at 48 h. In contrast, both ACL and BACL significantly delayed drug release, with only 63.1% and 61.4% of 5-ALA released at 2 h, respectively. The release reached a plateau at 12 h, resulting in cumulative releases of 79.1% (ACL) and 77.4% (BACL) at 24 h. Regarding CAT, free CAT displayed a 77.5% release at 24 h, whereas ACL and BACL exhibited much slower initial release (36.8% and 37.6% within the first 8 h), which gradually increased to 61.5% and 62.6% at 24 h.

Upon incubation with proteinase K, the enzymatic activity of all groups decreased relative to the untreated control (free CAT without proteinase K) [28]. Over time, the relative activities of free CAT, ACL, and BACL progressively declined. Notably, free CAT was significantly more susceptible to protease degradation than the liposomal formulations. After 8 h, the average relative activity of free CAT plummeted to 22.6%, whereas ACL and BACL retained considerably higher activities of 61.7% and 60.0%, respectively (Figure 3G).

The physical stability of BACL was monitored during storage at 4 °C by periodically measuring particle size, zeta potential, and PDI (Figure 3H). Over the 14-day storage period, both particle size and PDI exhibited a moderate increase, rising from 122.4 nm to 178.8 nm and from 0.189 to 0.335, respectively. Meanwhile, the zeta potential shifted from −12.3 mV to −7.07 mV. These results indicate that the liposomes remained physically stable for up to 14 days, with the optimal stability observed within the first 10 days.

### 3.2. Hypoxic Relief and Brain-Targeted Tumor Suppression

**Figure 4:**
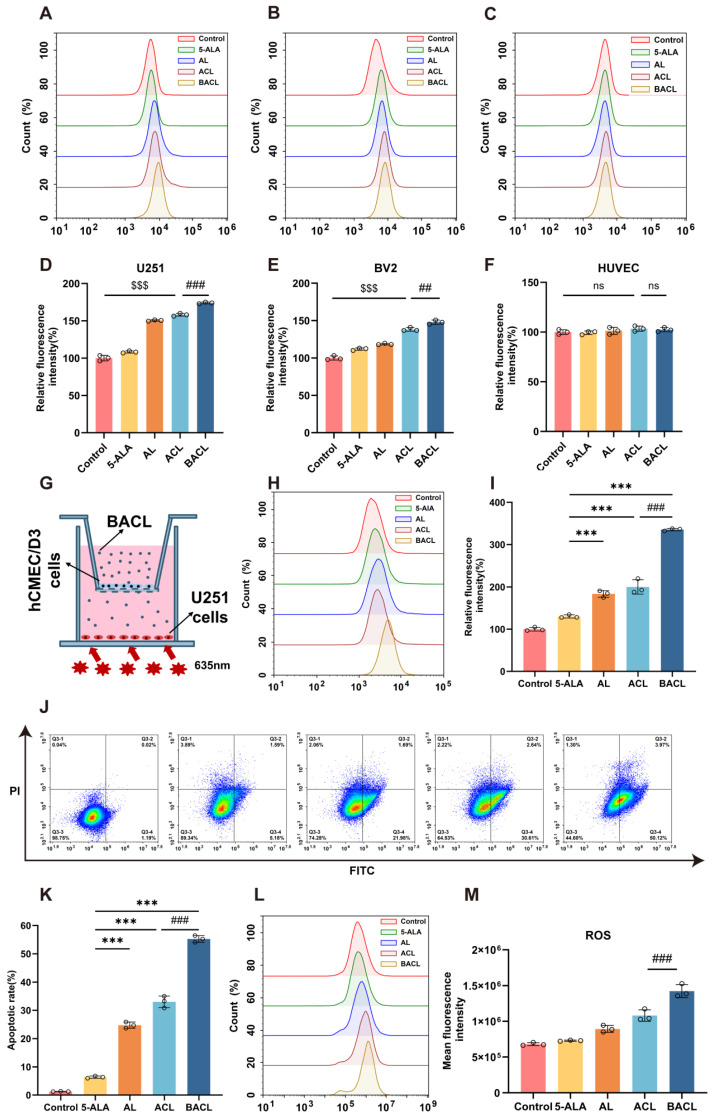
Results of hypoxia remission and brain-targeted tumor suppression. (**A**–**C**) Flow cytometry analysis of cellular uptake by U251 cells, BV2 cells and HUVECs. Images were captured using a 405 nm laser and a 660/20 nm emission filter. (**D**–**F**) Quantitative analysis of the mean fluorescence intensity from flow cytometry. (**G**–**I**) Cellular uptake of U251 cells incubated with different formulations using flow cytometry analysis ((**B**,**C**): hCMEC/D3 cell monolayer at 12 h; (**D**,**E**): U251 cell monolayer at 12 h). (**J**,**K**) Comparison of U251 cell apoptosis induced by different delivery systems, as quantified by flow cytometry. (**L**,**M**) Flow cytometry analysis of ROS by U251 cells. (Mean ± SD, *n* = 3, ^$$$^ *p* < 0.001, compared with the Control group; *** *p* < 0.001, compared with the 5-ALA group; ^##^ *p* < 0.01, ^###^ *p* < 0.001, ns, *p* > 0.05 compared with the ACL group).

Once internalized by cells, 5-ALA is converted into the photosensitive compound PpIX, which exhibits photosensitivity. The fluorescence intensity of PpIX within the cells was quantified using flow cytometry. The results indicated that Ang-2-targeted liposomes significantly enhanced the uptake of 5-ALA by GBM cells, achieving statistical significance. The brain-targeted uptake of BACL by U251 cells, BV2 cells, and HUVECs was further analyzed and compared. As shown in Figure 4A–F, the fluorescence intensity of PpIX in the experimental groups of U251 and BV2 cells exhibited an upward trend compared to the control group, with the fluorescence intensity in the BACL group significantly enhanced relative to that of the ACL group. However, no significant difference was observed in the uptake of ACL and BACL by HUVECs. Concurrently, imaging results from mice indicated that the brain fluorescence intensity in the BACL group was significantly higher than that in the ACL group after 6 h of administration (Figure S3).

To validate the effect of Angiopep-2 modification on enhancing BBB penetration of BACL, an in vitro BBB model was established. Flow cytometry analysis showed significantly higher intracellular accumulation in U251 cells treated with AL, ACL, and BACL compared to 5-ALA. Notably, Angiopep-2-mediated BACL exhibited superior targeted uptake over ACL (Figure 4G–I and Figure S4). In a transwell co-culture model assessing post-penetration efficacy, BACL, catalyzed by CAT, induced significantly more apoptosis in U251 cells and showed a stronger inhibitory effect than ACL and AL (Figure 4J,K).

To evaluate the effect of CAT in a hypoxic cell model, flow cytometry was employed to measure the fluorescence intensity of ROS in U251 cells after laser treatment. Compared with the control group, the relative fluorescence intensity of intracellular ROS was 1.07-fold in the 5-ALA group, increased to 1.59-fold in the ACL group, and further reached 2.09-fold in the BACL group (Figure 4L,M). These findings demonstrate that liposomes not only facilitated the transport of 5-ALA into tumor cells but also co-delivered CAT. The internalized CAT catalyzed the decomposition of H_2_O_2_ into O_2_ within the tumor’s hypoxic microenvironment, and O_2_ was subsequently converted into ROS under the influence of PpIX.

Analysis with the Image-iT™ Green Hypoxia Reagent revealed a significant increase in fluorescence intensity in the hypoxia group compared to the normoxia group (*p* < 0.001), confirming the successful establishment of a hypoxic microenvironment. Following drug intervention, both the ACL and BACL groups exhibited a reduction in the fluorescence signal, indicating an alleviation of hypoxia. Notably, the fluorescence intensity in the BACL group was significantly lower than that in the ACL group, demonstrating its superior hypoxia-relieving effect attributable to the efficient delivery and release of CAT (Figure S5). To further clarify the improvement effects of BACL and ACL on hypoxia in the tumor microenvironment, we detected the expression levels of HIF-1α protein in cells of each group by Western Blot. The results are shown in Supplementary Figure S6. Compared with the Control group, the expression of HIF-1α slightly decreased in the 5-ALA and AL groups; however, BACL and ACL treatments significantly reduced the expression level of HIF-1α (*p* < 0.01). These results indicate that both BACL and ACL successfully deliver CAT into tumor cells, thereby alleviating the hypoxic microenvironment of U251 cells. Notably, the effect of BACL was superior to that of ACL.

To investigate the molecular mechanism underlying BACL-mediated photodynamic therapy (PDT), Western blot analysis was performed to detect the expression of apoptosis-related proteins Bcl-2, BAX, and p53 (Figure S7). BACL significantly inhibited Bcl-2 expression (*p* < 0.001) while upregulating BAX and p53 (*p* < 0.05). These results demonstrate that BACL promotes apoptosis in GBM cells by modulating the Bcl-2/BAX/p53 pathway, contributing to its therapeutic efficacy.

### 3.3. In Vitro Therapeutic Effects

**Figure 5:**
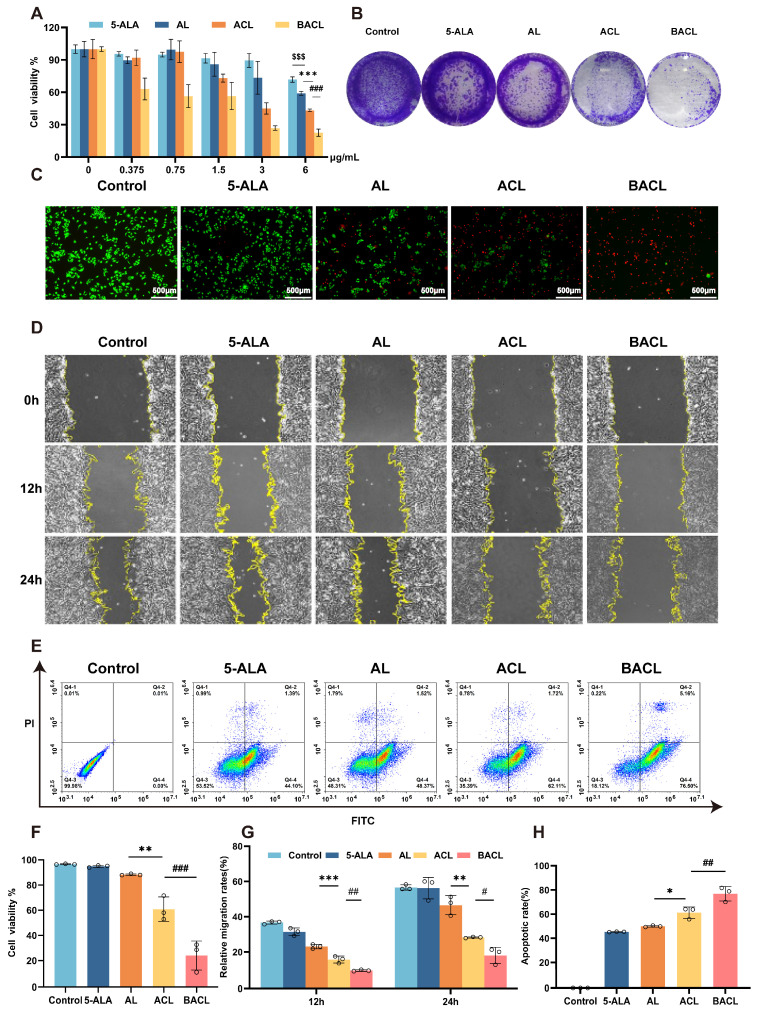
Illustrates the therapeutic efficacy and mechanism of BACL in vitro. (**A**) The cell viability of U251 cells was assessed using the MTT assay following treatment with various delivery systems at different concentrations over a 24-h period. (**B**) Results from the colony formation assay of U251 cells are presented. (**C**) Outcomes of Live and Dead cell staining are displayed. (**D**) Findings from the scratch healing experiment are detailed. (**E**) The effects of different delivery systems on apoptosis in U251 cells are evaluated. (**F**) Quantitative analysis of the Live and Dead cell staining. (**G**) Quantitative analysis of the scratch healing experiment. (**H**) Quantitative analysis of the apoptosis in U251 cells. (Data are presented as Mean ± SD, with statistical significance indicated as ^$$$^ *p* < 0.001 when compared with the 5-ALA group; * *p* < 0.05, ** *p* < 0.01, *** *p* < 0.001 when compared with the AL group; and ^#^ *p* < 0.05, ^##^ *p* < 0.01, ^###^ *p* < 0.001 when compared with the ACL group, *n* = 3 per group, scale bar: 500 μm).

The effects of 5-ALA, AL, ACL, and BACL on the viability of U251 cells under light conditions were evaluated using the MTT assay (Figure 5A). Concentration gradients of 5-ALA were selected at 0, 0.375, 0.75, 1.5, 3, and 6 μg/mL, with an incubation time of 24 h. The results demonstrated that as the concentration of 5-ALA increased, cell viability in each group decreased compared to the control group, indicating that 5-ALA, AL, ACL, and BACL all exhibited a significant inhibitory effect on tumor cell viability. When the concentration of 5-ALA reached 6.0 μg·mL^−1^, the cell viability in the BACL group was approximately 22.6%, significantly lower than that in the 5-ALA and ACL groups. The safety of the liposomal carrier was verified, as no significant difference in cell viability was observed between the blank liposome (Lip) group and the control group (Figure S8). Furthermore, under normoxic conditions, the anti-tumor efficacy of both ACL and BACL was markedly attenuated, with a less pronounced decline in cell viability compared to that under hypoxia (Figure S9). Taken together, these findings demonstrate that the liposomal carrier itself is non-toxic, the therapeutic effect stems mainly from the encapsulated 5-ALA, and the enhanced performance of BACL is attributable to the synergistic combination of its functional components.

A colony formation assay was performed to evaluate the effects of each treatment on cell proliferation (Figure 5B). Compared to the control group, all treatment groups exhibited varying reductions in colony number. Notably, the ACL group showed a significant decrease, while the BACL group displayed the fewest colonies among all groups. These findings suggest that incorporating 5-ALA and CAT into targeted liposomes substantially diminishes the proliferative capacity of U251 cells, and that CAT enhances the photodynamic inhibitory effect of 5-ALA on GBM.

To visually evaluate the anticancer effect in vitro, we employed a detection kit to stain and observe the cells treated with various preparations. As illustrated in Figure 5C,F, the BACL group exhibited a higher proportion of intracellular red fluorescence compared to the other groups, indicating that BACL significantly promoted GBM cell apoptosis. With prolonged incubation time, the targeted drug-carrying liposomes markedly inhibited the migration and invasion of U251 cells. The scratch assay results revealed notable reductions in *relative migration rates* of 50% and 68% for the ACL and BACL groups, respectively, compared to the control group (Figure 5D–G).

The Annexin V-FITC/PI apoptosis detection kit was used to quantitatively evaluate apoptosis in each group after laser treatment. As demonstrated in Figure 5E,H, the average apoptosis rates for the 5-ALA, AL, ACL, and BACL groups were 45.31%, 49.92%, 61.21%, and 76.86%, respectively, indicating an increasing trend in the proportion of GBM cells undergoing apoptosis. Notably, the BACL group exhibited the most pronounced apoptotic effect compared to the ACL group (*p* < 0.01), suggesting that brain-targeted liposomes effectively delivered CAT into cells and enhanced the photodynamic therapeutic effect of 5-ALA. These findings were consistent with the results obtained from the MTT assay.

### 3.4. In Vivo Efficacy and Biosafety

**Figure 6:**
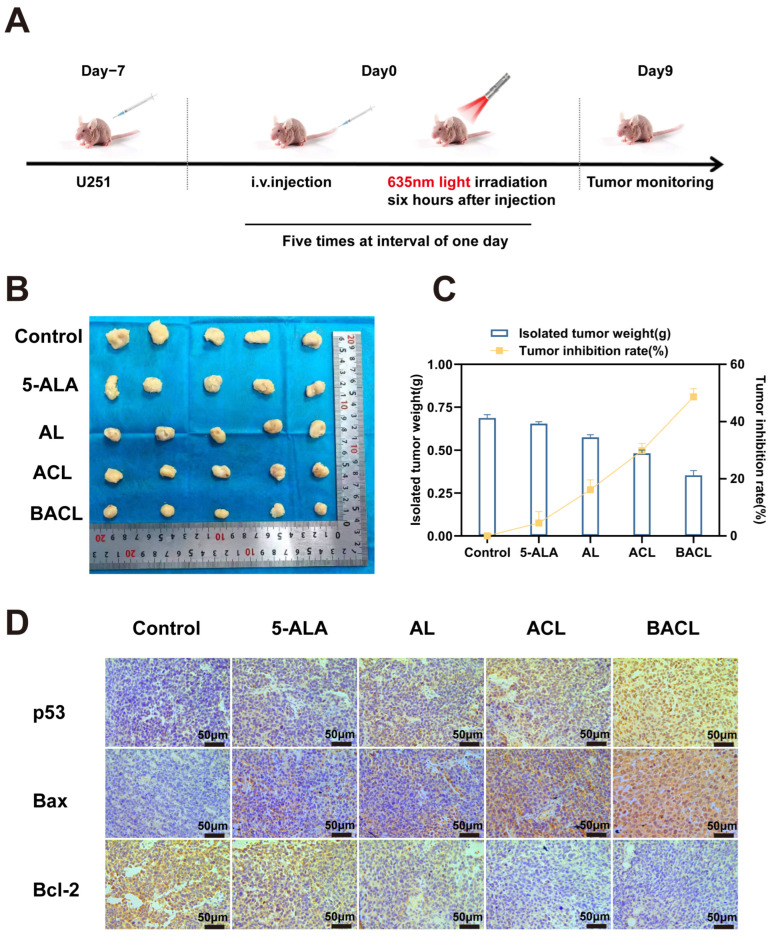
Evaluation of anti-tumor efficacy of BACL in vivo. (**A**) Schedule of the therapeutic procedure for BACL: administered every two days (40 mg/kg), followed by a 635 nm laser (0.3 W/cm^2^) 6 h post-administration. (**B**) Representative photographs of excised tumors from each treatment group. Tumors were harvested at the end of the study from mice treated with: (1) Control (PBS), (2) Free 5-ALA, (3) AL, (4) ACL, (5) BACL. (**C**) Tumor weight and inhibition rate curves for different treatment groups (*n* = 6). (**D**) Immunohistochemistry staining results for tumor tissue in each group following treatment (scale bar: 50 μm).

Local tumor irradiation was conducted according to the specified administration regimen, and changes in mouse body weight and tumor volume were systematically recorded throughout the treatment period (Figure 6A). The results indicated that mice in the control group experienced progressive weight loss attributable to tumor burden, whereas those in the liposome formulation groups (AL, ACL, BACL) maintained stable body weight, suggesting a low systemic toxicity associated with the formulations. All treatment groups demonstrated significant tumor growth inhibition, with the BACL group exhibiting the most pronounced effect, achieving a tumor inhibition rate of 52% (Figure 6B,C). Immunohistochemistry (IHC) was utilized to assess the expression of apoptosis-related proteins in tumor tissues. In alignment with the in vitro Western blot results, BACL treatment markedly upregulated the expression of the pro-apoptotic proteins p53 and BAX, while concurrently downregulating the expression of the anti-apoptotic protein Bcl-2 in GBM tissues (Figure 6D).

**Figure 7:**
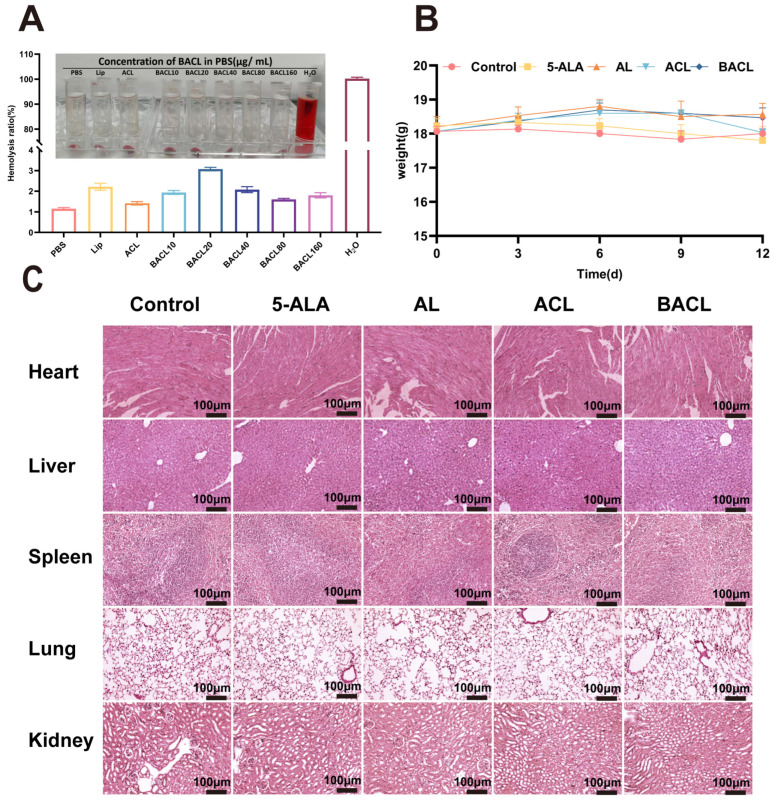
Evaluation Biosafety of BACL in vivo. (**A**) Hemolysis assay of preparation cocultured with rat blood cells (2% suspension in PBS) at 37 °C for 2 h. (**B**) Weight changes of mice in each group after drug administration. (**C**) HE staining results (heart, liver, spleen, lung, and kidney) for each group following treatment (Mean ± SD, *n* = 6, scale bar: 100 μm).

To assess the safety of intravenous administration, an in vitro hemolysis assay was performed. As shown in Figure 7A, both ACL and BACL exhibited no significant hemolytic effects across all tested concentrations, with hemolysis rates far below 5%, indicating excellent blood compatibility. Furthermore, after treatment with 40 mg/kg BACL, no significant difference in body weight was observed between the treated groups and the blank control group. H&E staining of major organs (heart, liver, spleen, lung, and kidney) revealed no obvious pathological abnormalities or inflammatory infiltration (Figure 7B,C).

### 3.5. Analysis of the Tumor Suppressor Mechanism of BACL

**Figure 8:**
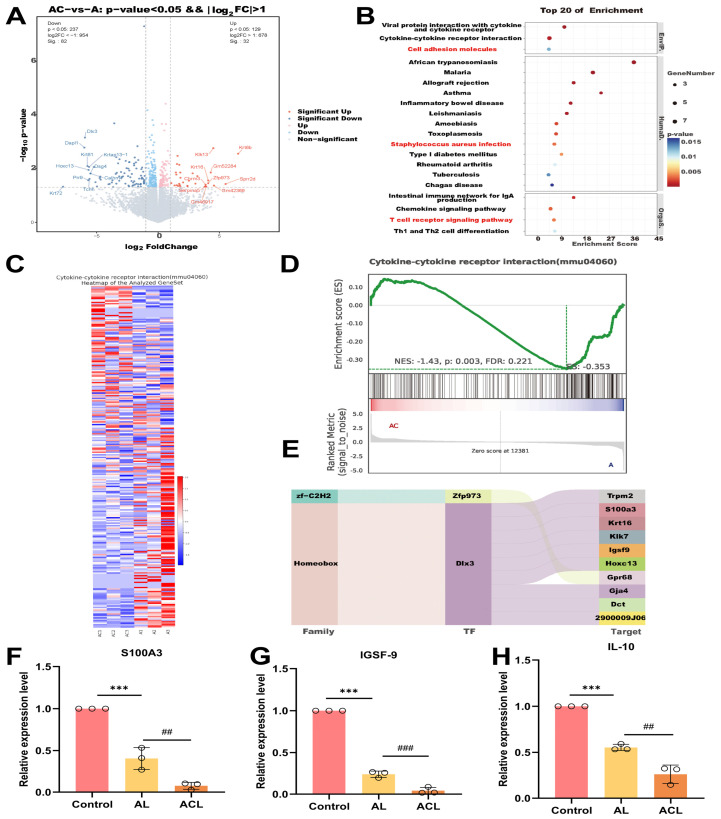
Analysis and validation of the tumor inhibitory mechanism of BACL. (**A**) Volcano plot displaying differentially expressed genes (DEGs) between ACL-treated (AC) and AL-treated (A) GBM highlighting genes with significant up-regulation (red) and down-regulation (blue) (cutoff: |log_2_FC| > 1, *p*-adjust < 0.05). (**B**) KEGG pathway enrichment analysis comparing ACL-treated GBM (AC) to AL GBM (A). (**C**) Heatmap visualizing the expression patterns of genes belonging to the ‘Cytokine–cytokine receptor interaction’ pathway across different treatment groups. (**D**) Gene Set Enrichment Analysis (GSEA) of regulated genes in ACL-treated (AC) GBM compared to AL (A) GBM. (**E**) Diagram of transcription factors and their target genes. (**F**–**H**) Quantitative RT-PCR validation of key differentially expressed genes in GBM tissues: (**F**) S100A3, (**G**) IGSF-9, and (**H**) IL-10. mRNA levels were normalized to GAPDH. (*** *p* < 0.001 compared to the control group; ^##^ *p* < 0.01; ^###^ *p* < 0.001 compared to the AL group).

To elucidate the mechanism of catalase (CAT) in 5-aminolevulinic acid (5-ALA) photodynamic therapy (PDT) for GBM, RNA was sequenced from two groups of tumor tissues: the AL- and ACL-treated groups (40 mg/kg). The volcano plot illustrates the differentially expressed genes between the two groups, with red indicating up-regulated genes and blue indicating down-regulated genes (Figure 8A). Differential gene enrichment analysis, including Gene Ontology (GO), Wiki Pathways, and KEGG enrichment analysis, revealed that ACLs significantly enriched cytokine–cytokine receptor interactions and negatively regulated interleukin-10 inflammatory signaling pathways compared to the AL group (Figure 8B and Figure S10A). Furthermore, gene set enrichment analysis (GSEA) confirmed that CAT could down-regulate the expression of IL-10, CXC chemokine ligand 13 (CXCL13), and other genes by modulating cytokine–cytokine receptor interactions, thereby enhancing the efficacy of 5-ALA photodynamic therapy for GBM (Figure 8C,D).

Analysis of differential transcription factors in the context of GBM and PDT further indicated that DIX3-regulated target genes were heavily enriched in pathways governing cytokine/immune regulation, cell adhesion molecules (CLDNs), and inflammation/angiogenesis, which collectively contribute to immunomodulation, tumor invasion, and the inflammatory/vascular niche in GBM, with S100A3 and IGSF-9 being key factors in cell adhesion and infiltration of immune cells (Figure 8E and Figure S10B–D).

To validate the RNA sequencing data, we evaluated the effects of 5-ALA combined with CAT liposomes (ACL) on gliomas using polymerase chain reaction (PCR). Interleukin-10 (IL-10) is a crucial immunomodulatory cytokine that promotes GBM growth and metastasis by stimulating angiogenesis and immunosuppression [29,30,31]. The expression of IL-10 was analyzed in mouse tumor tissues, and the PCR results indicated a downward trend in IL-10 expression in the ACL treatment group (Figure 8H), which aligned with the sequencing results. Subsequently, we assessed the target genes IGSF-9 and S100A3, which correspond to differential transcription factors, to investigate their roles in improving microenvironmental hypoxia and tumor prognosis [32,33]. Compared to AL, the combination of 5-ALA liposomes with CAT significantly inhibited the expression of S100A3 and IGSF-9 in GBM tissue, thereby enhancing the efficacy of 5-ALA photodynamic therapy (Figure 8F–H).

## 4. Discussion

Liposomes, as versatile nano-delivery systems, feature a bilayer vesicle structure capable of co-encapsulating hydrophilic agents within their aqueous core and lipophilic compounds within the lipid bilayer, while also accommodating biomacromolecules through surface modifications [34]. To overcome hypoxia-induced resistance in the tumor microenvironment during 5-aminolevulinic acid photodynamic therapy, we engineered liposomes for the dual delivery of 5-ALA and catalase (CAT). This approach utilizes the enzymatic function of CAT to convert endogenous H_2_O_2_ into molecular oxygen, which facilitates tumor oxygenation and improves the effectiveness of PDT. 5 ALA possesses both an amino group and a carboxyl group, and its ionization state is pH dependent. At the external phase (pH 8.0), 5 ALA exists predominantly in its molecular form, diffuses across the lipid bilayer into the internal aqueous phase, and is rapidly protonated into a cationic form, where it is retained inside the liposome lumen via an “ion trapping” effect [35]. Meanwhile, electrostatic interactions in the acidic internal environment further 623 enhance its retention stability. Our experiments showed that when the external phase pH was raised from 6.0 to 8.0, the encapsulation efficiency was significantly increased, confirming that a large pH gradient is the key factor driving efficient loading of 5 ALA. Furthermore, the thermal instability of CAT imposes specific requirements on the thermodynamic properties of the lipid membrane. To ensure the stability of the lipid bilayer during preparation, we selected the synthetic phospholipid PC70, which has a phase transition temperature above the processing temperature, over natural phospholipids like soy lecithin (S100, PC50) or egg yolk lecithin (E80) that have lower phase transition temperatures [33]. The resulting PC70-based liposomes demonstrated excellent encapsulation efficiency for CAT. This is owing to the higher saturation of PC70, which generally confers a thicker lipid bilayer structure that better facilitates the stable retention of high-molecular-weight biomacromolecules, such as CAT (molecular weight ≈ 240 kDa).

The hypoxic microenvironment of GBM not only promotes tumor progression but also severely limits the efficacy of photodynamic therapy (PDT) [36,37]. To tackle this challenge, we implemented a dual strategy: (i) CAT-mediated decomposition of hydrogen peroxide (H_2_O_2_) for self-oxygenation, and (ii) modification with DSPE-PEG_2000_-Ang2 for active targeting [37,38]. In comparison to non-targeted formulations (AL and ACL), Ang-2 modification significantly enhances the specific accumulation of 5-ALA in GBM through dual targeting of the blood–brain barrier (BBB) and GBM, thereby improving PDT efficacy while minimizing off-target toxicity.

Biocompatibility and biosafety are essential prerequisites for the clinical translation of nano-delivery systems [39,40]. Comprehensive biosafety evaluation data, derived from both in vitro and in vivo studies, collectively demonstrate the enhanced biocompatibility of this liposome delivery system. MTT assay results indicated that cell viability remained above 85% in the treatment concentration group under light-avoiding conditions. Systematic histopathological assessment of major organs revealed preserved tissue architecture, with no treatment-related pathological changes observed. These safety parameters provide a robust foundation for future clinical translation. Importantly, surface functionalization with DSPE-PEG_2000_ confers an invisibility property to the nanoparticles. This modification significantly reduces non-specific uptake by the reticuloendothelial system (RES) and effectively mitigates particle aggregation via steric hindrance effects [41]. Together, these mechanisms contribute to the favorable safety profile of the nanoparticles in terms of pharmacokinetic performance and physical stability. This favorable safety profile, together with the complementary evidence chain of “barrier penetration to tumor killing to dose optimization” established by the subcutaneous glioma model and in vitro BBB model in this study, collectively enhances the translational potential of this delivery system for neuro-oncological therapy.

5-ALA-basedPDT induces apoptosis in cancer cells through various mechanisms, including ROS-mediated mitochondrial dysfunction, vascular damage, and the stimulation of anti-tumor immunity via pro-inflammatory cytokines such as IL-6 and TNF-α [42]. Clinically, 5-ALA-guided fluorescence microsurgery achieves a total resection rate of 76.8% in patients with high-grade GBM, significantly surpassing the 42.1% rate observed with conventional white-light microsurgery (*p* < 0.001) [43]. These findings highlight the translational potential of 5-ALA in the field of neuro-oncology. At the clinical translation level, the choice of light delivery modality is equally critical. In the process of clinical translation, another key aspect of photodynamic therapy is the choice of light delivery modality.

Since 635 nm light penetrates only 2–5 mm into brain tissue, non-invasive transcranial irradiation is insufficient to reach deep-seated tumors. Therefore, intraoperative photodynamic therapy (PDT) is currently the preferred clinical approach: the optical fiber tip is placed on the surface of the surgical cavity or inserted into residual tumor tissue for localized irradiation [44]. This method bypasses the skull barrier and provides the rationale for using a subcutaneous tumor model as a proof-of-concept in this study. In addition, stereotactically guided interstitial photodynamic therapy (PDT) has been adopted clinically, wherein single or multiple fiber diffusers are implanted percutaneously under navigation to cover the tumor volume for treating recurrent glioblastoma (GBM) in deep or eloquent areas [45,46]. Combining these light delivery techniques with the brain-targeted nanocarriers developed in this study may enable precise photosensitizer delivery and spatial coverage of light dose, thereby improving the efficacy against deep-seated tumors, reducing damage to normal brain tissue, and laying the foundation for subsequent studies using orthotopic intracranial models. These findings suggest that the synergistic effect of light delivery strategies and nanomedicines warrants further exploration of their anti-tumor mechanisms at the molecular level.

Transcriptomic sequencing and bioinformatics analyses indicate that CAT combination therapy exerts anti-GBM effects by modulating the IL-10 signaling pathway, chemokine activities, and immune response mechanisms, while also reducing the expression of IGSF-9 and S100A3. Importantly, PDT-induced oxygen consumption may exacerbate tumor hypoxia, leading to the upregulation of IGSF-9, a gene associated with tumor growth, migration, and invasion [47,48,49]. Additionally, as a member of the calcium-binding S100 protein family, S100A3 has been implicated in the progression of low-grade GBM (LGG) through its modulation of the inflammatory microenvironment and immune cell infiltration [50].

Despite the encouraging findings of this study, several limitations warrant consideration. For instance, the understanding of the roles played by key factors such as S100A3 and IGSF-9 in cell adhesion and therapeutic efficacy is currently based primarily on bioinformatic analyses. Their precise molecular regulatory pathways still require further elucidation through experimental approaches such as targeted gene knockout or knockdown. Nevertheless, these limitations do not undermine the core conclusion of this study: the ACL formulation significantly enhances the efficacy of 5-ALA-based photodynamic therapy in experimental models. This work is the first to reveal that the combined use of 5-ALA and CAT can modulate IGSF-9 and S100A3, offering a novel nanotherapeutic strategy for improving the immune microenvironment in LGG. Future research will focus on elucidating the specific molecular mechanisms of 5-ALA to accelerate its clinical translation.

## 5. Conclusions

In this study, we developed brain-targeted 5-ALA-CAT Liposomes (BACL) designed to deliver both 5-ALA and catalase, addressing the challenges associated with poor photodynamic therapy (PDT) due to low permeability and the hypoxic microenvironment inherent to 5-ALA targeting. The results demonstrated that BACL significantly enhanced the efficacy of PDT in the treatment of GBM at cellular, tissue, and in vivo levels. The tumor inhibition rate achieved with BACL was 52%, without notable damage to major organs. Mechanistic verification revealed that BACL regulates GBM by modulating the expression of p53, BAX, and Bcl-2 proteins, while inhibiting the expression of IL-10, S100A3, and IGSF-9 genes. This study enhances the photodynamic tumor inhibition effect by improving both the hypoxic microenvironment and targeted delivery, providing new hope for advancing 5-ALA photodynamic diagnosis and treatment and expanding its application field.

## Data Availability

The raw data supporting the conclusions of this article will be made available by the authors upon reasonable request.

## Supplementary Materials

The following supporting information can be downloaded at: https://www.mdpi.com/article/10.3390/pharmaceutics18070777/s1, Figure S1: Serum stability of BACL in 50% FBS; Figure S2: Information on in vitro content determination of 5-ALA and CAT; Figure S3: Liposome biodistribution and quantitative brain targeting; Figure S4: Evaluation of in vitro BBB permeability and PDT effect; Figure S5: Image of Hypoxia status of U251 cells detected by Image-IT™ Green Hypoxia Reagent; Figure S6: The protein bands and relative expression level of HIF-1α; Figure S7: The protein bands and relative expression level of BAX, Bcl-2 and p53; Figure S8: The MTT analysis results of blank liposomes; Figure S9: Analysis of MTT results under normoxic conditions; Figure S10: Analysis and validation of the tumor inhibitory mechanism of BACL; Table S1: Sequence of primers; Table S2: Screening of trehalose dosage; Table S3: Stability of lyophilized product at room temperature (25 °C).

## Abbreviations

The following abbreviations are used in this manuscript:

5-ALA	5-aminolevulinic acid
BACL	Ang2-modified liposomes
BBB	Blood–brain barrier
BCL	brain-targeted CAT liposomes
CAT	Catalase
DEGs	Differentially expressed genes
EE	Entrapment efficiency
GBM	Glioblastoma multiforme
GSEA	Gene Set Enrichment Analysis
KEGG	Kyoto Encyclopedia of Genes and Genomes
LC	Loading capacity
MFI	Mean fluorescence intensity
PI	Propidium iodide
PpIX	Photosensitizer protoporphyrin IX
PPI	Protein–protein interaction
RBCs	Red blood cells
ROS	Reactive oxygen species
TEER	Trans endothelial electrical resistance
