# Supplementary material for: Brain-Targeted 5-ALA-CAT Liposomes (BACL) Alleviate Hypoxia and Enhance Photodynamic Therapy in a Murine Glioblastoma Flank Xenograft Model via Angiopep-2-Mediated Targeting
Source: Pharmaceutics. 2026 Jun 25;18(7):777. doi: 10.3390/pharmaceutics18070777 (PMC13414474; doi:10.3390/pharmaceutics18070777)
Supplement: Supplementary file 1 [file pharmaceutics-18-00777-s001.zip › pharmaceutics-4327500-supplementary.pdf]

pharmaceutics

Article

Brain-Targeted 5-ALA-CAT Liposomes (BACL) Alleviate Hypoxia and Enhance Photodynamic Therapy in a Murine Glioblastoma Flank Xenograft Model via Angiopep-2-Mediated Targeting

Qian Zhang [1,2,3], Yuhang Li [1,2,3], Jiahui Zhang [1,2], Xuewen Zhao [1,2], Danlu Li [1], Wenting Zhao [1], Xin Hai [1,3], Xin Chen [2], Xinlei Yang [4], Jingxin Gou [5], Chunpeng Zhang [1,2], Xing Tang [5,*] and Yilei Zhao [1,2,3,*]

1 Department of Pharmacy, The First Affiliated Hospital of Harbin Medical University, Harbin 150001, China; 19829136728@163.com (Q.Z.); li1324307746@163.com (Y.L.); 15164485820@163.com (J.Z.); zhaoxw5571@163.com (X.Z.); yxlidanlu@126.com (D.L.); zwting1986@163.com (W.Z.); hai_xin@163.com (X.H.); zhangcp1796@163.com (C.Z.)
2 Department of Neurosurgical Laboratory, The First Affiliated Hospital of Harbin Medical University, Harbin 150001, China; chenxin_tracy@yeah.net
3 Heilongjiang Province Key Laboratory of Precision Pharmaceutical Research, The First Affiliated Hospital of Harbin Medical University, Harbin 150001, China
4 Animal Laboratory Center, The First Affiliated Hospital of Harbin Medical University, Harbin 150001, China; xinleiyang@hotmai.com
5 Department of Pharmaceutics, School of Pharmacy, Shenyang Pharmaceutical University, Shenyang 110016, China; jxgou_syphu@163.com
* Correspondence: tangpharm@163.com (X.T.); zhaoyilei2014@126.com (Y.Z.)

1. Supplementary Figures and Tables

1.1 Supplementary Figures

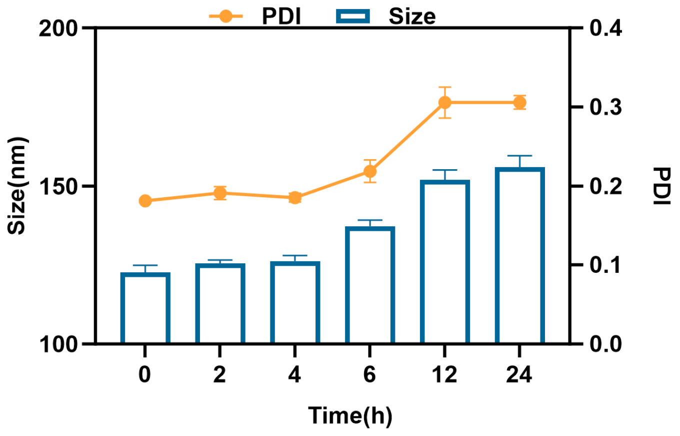

Figure S1. Serum stability of BACL in 50% FBS.

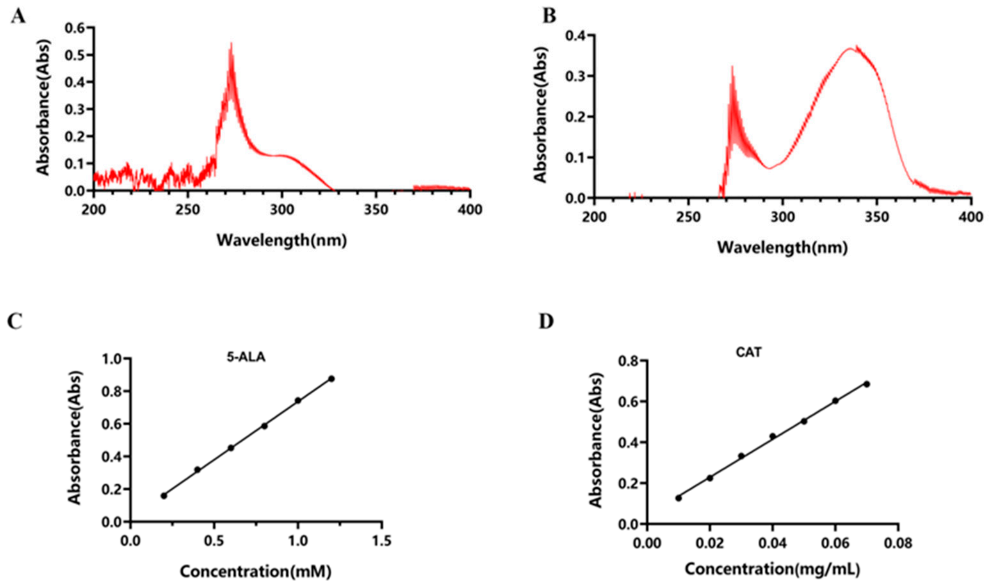

Figure S2. Information on in vitro content determination of 5-ALA and CAT. (**A**)The UV-vis spectrum of blank liposome. (**B**)TheUV-visspectrumof5-ALA. (**C**) The standard curve and linear fitted equation of 5-ALA (A = 0.713C + 0.0234 R^2 = 0.9992). (**D**) The standard curve and linear fitted equation of CAT (A = 9.2929C + 0.0436R^2 = 0.9977).

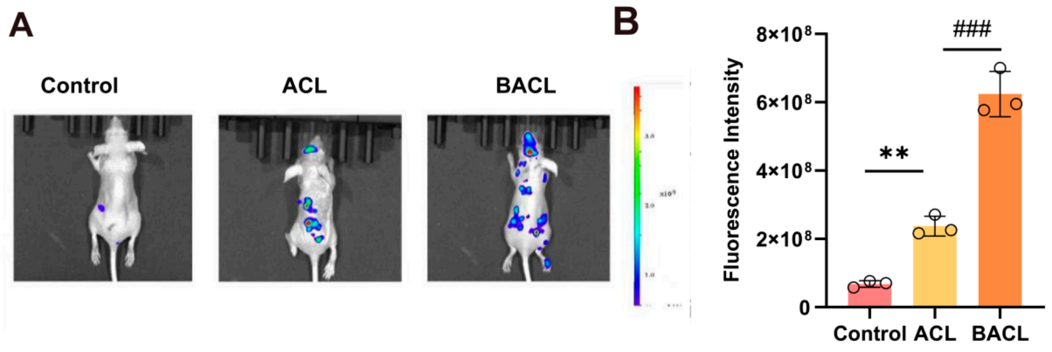

Figure S3. Liposome biodistribution and quantitative brain targeting. (**A**) In vivo fluorescence imaging at 6 h post-injection. (**B**) Quantitative analysis fluorescence intensity in mice brains following the DIO-labeled liposome at 6 h. (Mean ± SD, n = 3, $^{**}p$< 0.01, compared with the Control group; $^{###}p$ < 0.001， compared with the ACL group)

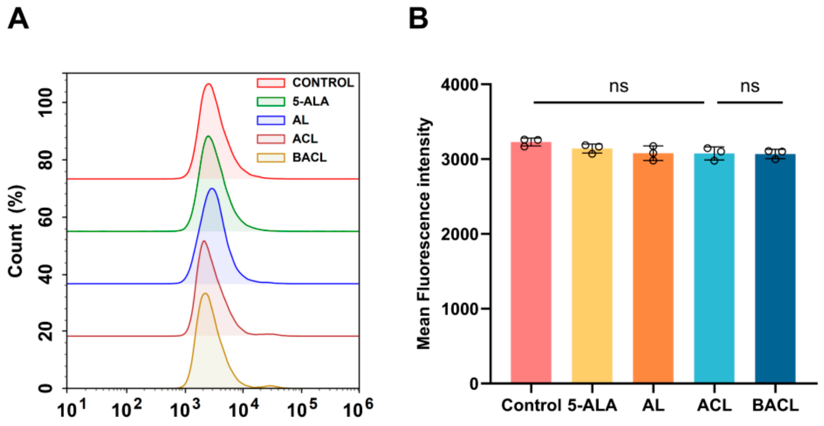

Figure S4. Evaluation of in vitro BBB permeability and PDT effect. (**A**) Cellular uptake of U251 cells incubated with different formulations using flow cytometry analysis (hCMEC/D3 cell monolayer at 12 h). (**B**)Quantitative analysis of the mean fluorescence intensity from flow cytometry. (Mean ± SD, n = 3, ns, p > 0.05 compared the ACL group)

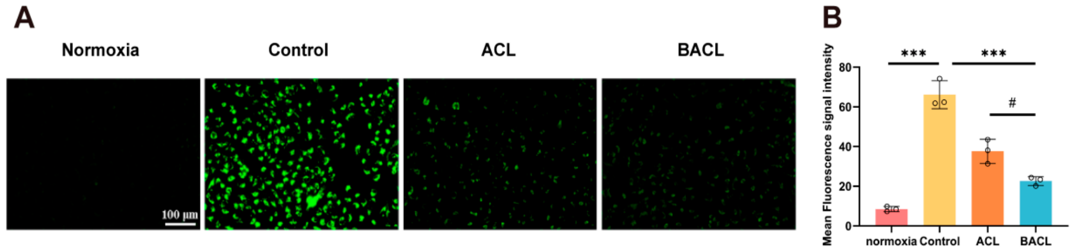

Figure S5. (**A**) Image of Hypoxia status of U251 cells detected by Image-IT™ Green Hypoxia Reagent. (**B**) Statistical analysis chart of hypoxia state. (Mean ± SD, n = 3，$^{***}p < 0.001$， compared with the Control group；$^{\#}p < 0.05$, compared with the ACL group)

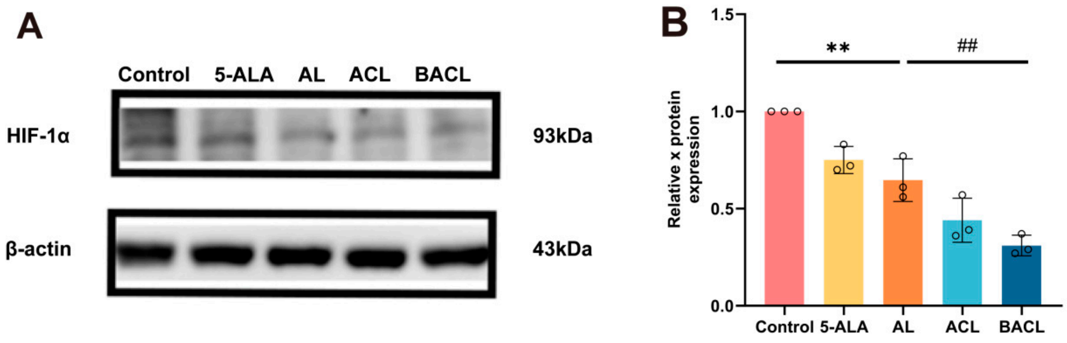

Figure S6. (**A**)The protein bands and relative expression level of HIF-1α. (**B**) The relative expression level of HIF-1α (Mean ± SD, n = 3, $^{**}p < 0.01$， compared with the Control group，$^{\#\#}p < 0.01$, compared with the AL group).

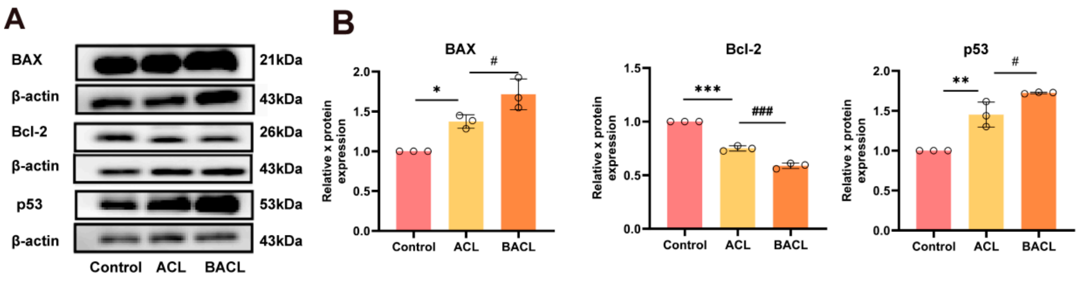

Figure S7 (**A**) The protein bands and relative expression level of BAX, Bcl-2 and p53. (**B**) The relative expression level of BAX, Bcl-2 and p53. (Mean ± SD, n = 3, $^{*}p < 0.05$, $^{**}p < 0.01$, $^{***}p < 0.001$, compared with the Control group; $^{\#}p < 0.05$, $^{\#\#\#}p < 0.001$, compared with the ACL group)

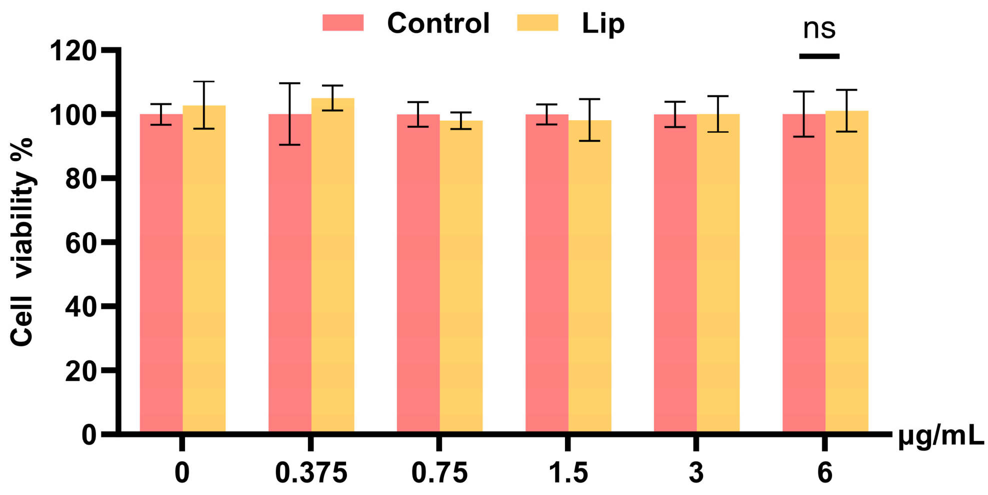

Figure S8 The MTT analysis results of blank liposomes. Data are presented as mean ± SD (n = 3, ns, $p > 0.05$).

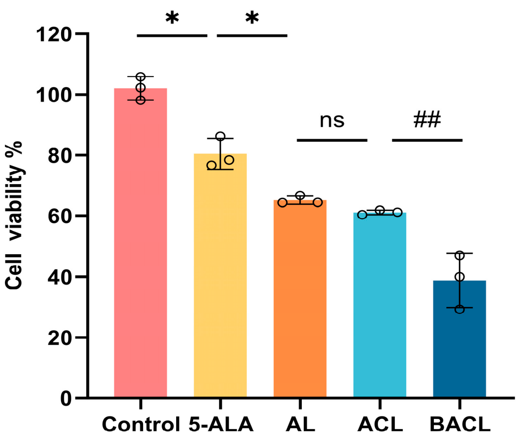

Figure S9 Analysis of MTT results under normoxic conditions. (Mean ± SD, n = 3, $^{*}p < 0.05$, compared with the 5-ALA group, $^{\#\#}p < 0.01$, compared with the ACL group, ns, $p > 0.05$ compared the ACL group)

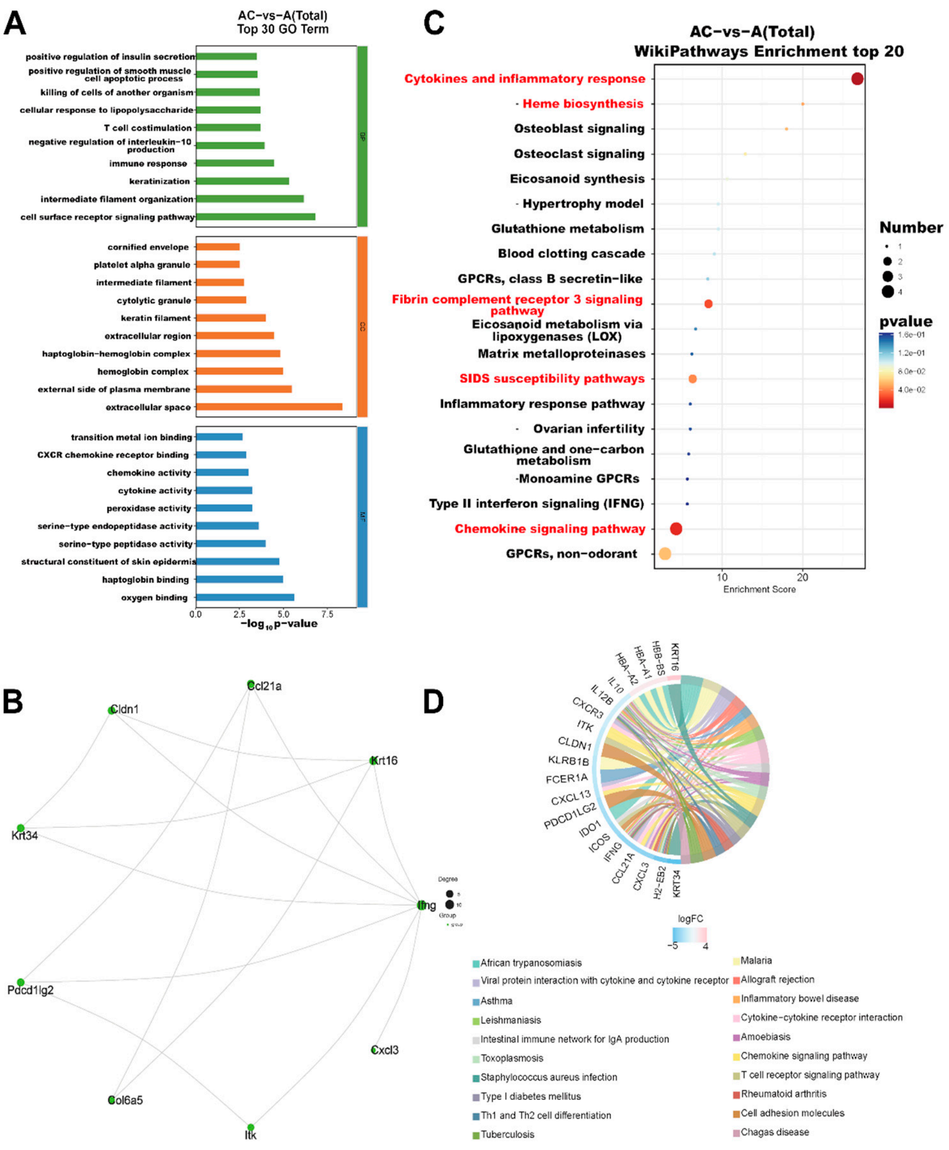

Figure S10. Analysis and validation of the tumor inhibitory mechanism of BACL. (**A**) Gene Ontology (GO) functional analysis of differentially expressed genes identified from the comparison of ACL-treated (AC) and AL-treated (A) gliomas. (**B**) Protein–Protein Interaction (PPI) Network of Differentially Expressed Genes (DEGs). (**C**) Wiki pathways enrichment analysis of significant pathways in AC vs. A gliomas. (**D**) Regulatory network between key transcription factors and signaling pathways.

1.2 Supplementary Tables

Table S1. Sequence of primers

	FORWARDSequence (5′–3′)	REVERSESequence (5′–3′)
S100A3	CTGAGTTTCGGGAATGTGACTACAAC	GAGACAGAGGCAGGCAAGTGAG
IGSF-9	CGCCTCCGCCAAGATCCAC	CGCCACGCTGTCAGGACTG

IL-10	**TTGCTGGAG-GACTTTAAGGGTTACC**	**TTGATGTCTGGGTCTT-GGTTCTCAG**
GAPDH	**GTGGACCTGAC-CTGCCGTCTAG**	**GAGTGGGTGTCGCTGTTGAAGTC**

Table S2. Screening of trehalose dosage

Treha-lose	Appearance	Reconstitu-tion time（s）	Particle size before ly-ophilization (nm)	Particle size after ly-ophilization (nm)	PDI before lyophiliza-tion	PDI after ly-ophilization
0.5%	Slight crystal precipitation at the bottom	18.7	129.4±1.01	368. 7±3.89	0.259±0.031	0.372±0.016
1%	Partial cak-ing/aggrega-tion	14.8	126.9±0.96	355.3±2.05	0.255±0.102	0.329±0.019
3%	Bottle shrink-age/collapse	10.6	124.4±1.02	262.4±1.12	0.230±0.041	0.319±0.011
5%	Bottle shrink-age/collapse	12.4	123.6±0.96.	258.7±1.69	0.238±0.035	0.295±0.035
10%	Porous and loose structure	10.4	121.3±0.82	136.5±2.34	0.217±0.022	0.228±0.023

Table S3. Stability of lyophilized product at room temperature (25 °C)

Time（day）	Particle size (nm)	PDI
1	136.9±1.29	0.228±0.023
5	139.2±1.34	0.221±0.048
10	142.4±1.02	0.239±0.041
20	144.8±1.57.	0.248±0.039
30	146.7±1.45	0.250±0.042
